# Commentary on strategies for switching antipsychotics

**DOI:** 10.1186/1741-7015-6-18

**Published:** 2008-06-30

**Authors:** John M Davis, Stefan Leucht

**Affiliations:** 1 Department of Psychiatry, University of Illinois at Chicago, 1601 West Taylor Street, Chicago, IL 60612, USA; 2 Department of Psychiatry and Psychotherapy, Technische Universität München, Klinikum rechts der Isar, Ismaningerstrasse, 81675 Munich, Germany

## Abstract

Both the new generation of antipsychotics and the more traditional antipsychotic drugs produce an important and meaningful improvement in patients with schizophrenia, but most patients are neither cured nor free of symptoms. As a consequence, it is common to switch from one drug to another in the hope of obtaining a better response. All antipsychotic drugs produce some side effects, so switching can also be a tolerance issue. There are reports in the literature on the tactics of switching: abrupt discontinuation, cross tapering, starting a patient on a new drug while continuing with the old drug until the new drug has reached a steady state, or some variation on these tactics. In this issue, Ganguli et al. have carried out a randomized switching study, the data from which indicates the tactics that might be optimal. We put this paper into context, provide a critique and describe indications for switching.

## Background

Ganguli et al. [1] studied the strategy of switching patients from olanzapine to risperidone. They found that slow tapering of the initial antipsychotic after the new drug had been titrated to the full dose produces fewer problems during the switch than abrupt discontinuation or gradual discontinuation before starting a new drug.

The abrupt discontinuation of clozapine does produce an acute worsening of psychosis in some patients and side effects as a result of withdrawal [2]. It is possible that some withdrawal side effects arise from the discontinuation of antipsychotic drugs and/or the antiparkinsonian drugs (often co-administered to reduce extrapyramidal side effects). It is less clear whether this occurs with other antipsychotic drugs [2]. Olanzapine has many pharmacological similarities to clozapine, so it is plausible that such phenomena could occur. Problems that might occur on switching include rebound worsening of psychotic symptoms, side effects, such as the addition of side effects of the old and new drugs, or side effects specific to the new drug, or differences in efficacy between the drugs. Problems might be specific to the discontinuation of the drug or to the drug to which the patient is switched. This strategy (sometimes called 'overlap and taper') ensures that the patient is covered with an adequate plasma level of the added drug before the former drug is discontinued. An increase in side effects did not occur in the Ganguli et al. study [1] or in other studies [3-8]. Such a strategy would not apply to all drugs where the side effects of each of the drugs could combine to produce a significant increase in side effects.

## Discussion

Knowing the tactics of switching advances the practical use of these drugs and advances the field. We have good basic information on efficacy, safety and pharmacokinetics from registrational studies. However, there is little information on many of the practical and strategic issues of drug administration [9]. Ganguli et al.'s study is one step towards remedying the situation. We would distinguish this study, which makes a clear-cut contribution, from many of the post-marketing studies supported by the pharmaceutical industry, which merely replicate efficacy and safety studies that have been performed many times before. The endless replication of similar studies does not add much information, but studies directed at filling in the gaps in our knowledge do. In our opinion, some of the promotional material exaggerates the possibilities of withdrawal effects.

### Role of open studies

Studies on the switch process are also carried out in part for marketing purposes to show that there is improvement on the new drug. We have shown that open studies systematically produce more positive results favoring the sponsor and are pertinent to the evaluation of such studies. This said, such open studies are valuable: it is harder to recruit patients (or to recruit physicians to manage patients) in double-blind studies than in open studies. Some relaxation of the controls against bias is traded off against the lower costs and more representative samples of patients. Some flexibility in the design of studies on the tactics of using drugs will result in a wider range of studies being carried out at an affordable cost.

### Weight gain: the most common reason to switch

The most common reason for switching from olanzapine is excessive weight gain. Antipsychotic drugs differ in their propensity to cause weight gain: olanzapine and clozapine cause the most; quetiapine and risperidone cause some; ziprasidone, aripiprazole, amisulpride and haloperidol cause little or no weight gain. There are wide individual differences among patients on a given drug: some patients gain substantial weight, others gain little, some gain none and some lose weight. The time course of weight gain is fairly rapid in the first few weeks, but slows down and seems to plateau after several months. Young patients (that is, children, adolescents and young adults) are much more liable to gain a substantial amount of weight. The rank ordering of antipsychotic weight gain propensity is similar in younger patients; however, even haloperidol causes substantial weight gain among young patients. Patients who had previously been on a high weight-gaining antipsychotic are much less liable to gain more than those who were not. It is important, therefore, to weigh patients approximately every week in the first few months of their treatment and then less frequently afterwards. Substantial weight gain is associated with type-2 diabetes and, for some patients, the consequences of obesity must be regarded as a serious medical event. If it is apparent that a given patient is likely to gain a lot of weight, the clinician is faced with limiting weight gain by diet, exercise, a pharmacological intervention or by switching to another drug, which causes less weight gain. As most of the weight gain occurs early on, the switch or other coping strategies should be made as soon as possible. Therefore, the problem of weight gain should be faced in the first 3 to 6 weeks of drug treatment and a strategy (diet, exercise, a concomitant drug, which might reduce weight gain, or switching antipsychotics) should be devised to limit the weight gain rather than wait until the patient has already gained weight when it is hard to lose.

### To switch or not to switch

Although antipsychotic administration produces considerable benefits in schizophrenic patients, these drugs do not produce a complete or permanent cure. Many residual symptoms remain and continued drug use is required, essentially for life, to prevent relapse. Since patients do have residual symptoms, there is hope that a better response could be achieved with a different drug. Consequently, switching or the addition of other augmenting drugs occurs frequently. The various antipsychotics differ in efficacy, side-effect profiles and cost. Not every patient has every side effect. It is likely that one could find, by trial and error, a drug with a better side-effect profile for an individual patient. There is evidence from controlled trials that patients who do not tolerate or like a given drug will neither like nor tolerate that drug when randomized in a double-blind randomized-assignment trial [10]. It is not known whether there are individual differences such that one patient will respond to drug A and not drug B; another to drug B but not drug A. A past history of a patient's experience with a given drug can be helpful in the choice of drug. Thorough documentation of the dose, side effects and therapeutic efficacy will facilitate changes in medication in the future.

## Conclusion

In general, there are few problems with switching from one antipsychotic to another, but they do occur. In deciding to switch, Ganguli et al.'s study suggests that maintaining the full dose of the initial antipsychotic until the second drug has been titrated to its full dose is the preferred strategy.

## Pre-publication history

The pre-publication history for this paper can be accessed here:


